# Nut Cracking Tools Used by Captive Chimpanzees (*Pan troglodytes*) and Their Comparison with Early Stone Age Percussive Artefacts from Olduvai Gorge

**DOI:** 10.1371/journal.pone.0166788

**Published:** 2016-11-21

**Authors:** Adrián Arroyo, Satoshi Hirata, Tetsuro Matsuzawa, Ignacio de la Torre

**Affiliations:** 1 Institute of Archaeology, University College London, 31–34 Gordon Square, London, WC1H 0PY, United Kingdom; 2 Kumamoto Sanctuary, Wildlife Research Center, Kyoto University, 990 Ohtao, Misumi, Uki, Kumamoto, 869–201, Japan; 3 Kyoto University, Institute for Advanced Studies, Kyoto, Japan; 4 Primate Research Institute, Kyoto University, Aichi, Japan; 5 Japan Monkey Centre, Aichi, Japan; Max Planck Institute for the Science of Human History, GERMANY

## Abstract

We present the results of a series of experiments at the Kumamoto Sanctuary in Japan, in which captive chimpanzees (*Pan troglodytes*) performed several nut cracking sessions using raw materials from Olduvai Gorge, Tanzania. We examined captive chimpanzee pounding tools using a combination of technological analysis, use-wear distribution, and micro-wear analysis. Our results show specific patterns of use-wear distribution across the active surfaces of pounding tools, which reveal some similarities with traces on archaeological percussive objects from the Early Stone Age, and are consistent with traces on other experimental pounding tools used by modern humans. The approach used in this study may help to stablish a framework with which to interpret archaeological assemblages and improve understanding of use-wear formation processes on pounding tools used by chimpanzees. This study represents the first direct comparison of chimpanzee pounding tools and archaeological material, and thus may contribute to a better understanding of hominin percussive activities.

## Introduction

Between 6 and 8 million years ago, the extinct relatives of chimpanzees (*Pan*) and hominins shared a common ancestor [1] who probably used unmodified stones as tools [2]. This has important evolutionary implications, especially following the recent discovery of the earliest stone tools (Lomekwian) at Lomekwi 3 (West Turkana, Kenya), which has pushed back the dawn of stone flaking to 3.3 Mya [3]. The Lomekwi assemblage shows that hominins were intentionally flaking stone tools, and suggests the emergence of stone tool knapping during a period close to the divergence of panin and hominin lineages. Therefore, stone flaking might not be associated exclusively with the genus *Homo*, but may have occurred in other taxa. At 3.3 Mya, the only hominins known (and therefore the likely makers of the Lomekwian tools) had brains no bigger than living African apes. Thus, comparison of hominin and chimpanzee products of behaviour are apt, and the use of living non-human primates as an analogy on which to model and understand earlier stages of human evolution, has become more relevant than ever [4–9].

It has been suggested that prior to the appearance of stone tool knapping, hominins probably used various organic tools that are archaeologically invisible [2, 10]. The use of pounding tools is a common behaviour recognized in both living non-human primates and the archaeological record. Pounding activities could have played an important role in hominin behaviour and probably contributed to the emergence of stone tool knapping [11–13]; the latter is a major research topic in the archaeology of human evolution and the subject of much debate.

Research in recent decades has focused on the use of chimpanzees as a reference to model hominin behaviour and the emergence of stone knapping. However, the models generated have limitations, as they directly compare flaked archaeological tools with pounding tools used in chimpanzee nut cracking activities. As the archaeological record also contains pounding tools that can be compared with battered artefacts produced by modern primates, it is possible to develop cross-disciplinary comparative frameworks.

This study provided a unique opportunity to develop a comparative approach, as it combined primatological behavioural observations and analysis of stone tools used by captive chimpanzees. We collected raw materials at Olduvai Gorge (Tanzania) and conducted a series of experiments with captive chimpanzees at the Kumamoto Sanctuary (Japan). We used techno-typological descriptions and low magnification microscopic and use-wear spatial distribution analyses of captive chimpanzee experimental artefacts (see below). The results can be directly compared with findings from other studies on modern humans [14] and wild chimpanzees [15], and contribute to the creation of a larger dataset with which to better understand the role of percussive activities in human evolution.

### Background

Long-term primatological field observations have enabled the study of nut cracking in wild chimpanzees (*Pan troglodytes*), an activity that infant individuals (of about 3.5 years old) begin to learn by observing their mothers and other members of the community in a process that Matsuzawa et al. named ‘*education by master-apprenticeship’* [16–19].

Field observations of wild chimpanzees reveal sex differences in nut cracking performance [20, 21], variation between neighbouring groups [22], and shed light on stone tool transport [23, 24] and raw material selection [25]. Studies have also revealed that West African chimpanzees use a range of tools to process nuts, including clubs, mobile anvils and hammers, fixed anvils available in the landscape (roots), and branches [26–28]. As such, pounding activities are part of a much wider tool-using repertoire defined as complex cultural behaviour [29].

In addition, some captive chimpanzees research projects have studied the motion and kinetics involved in nut cracking activities [30, 31], and those aspects that reflect chimpanzee cognitive capabilities (e.g. object manipulation, transmission of tool use, and colour recognition) [32, 33]. Others studies have focused on behavioural patterns and the process of learning to crack nuts using a hammer and anvil [34–37].

Wynn and McGrew ([4], page 384) stated that ‘the most direct way to compare Oldowan and chimpanzee technology is to compare the tools themselves’, and argued that there are similarities in hominin and chimpanzee tool use. Mercader and colleagues [7] claimed similarities between the by-products found at the Panda 100 chimpanzee nut cracking site and Oldowan flakes. Recently, research has focused on reconstruction of the *chaîne opératoire* of nut cracking [24] and the application of 3D and geographic information system (GIS) techniques in the analysis of chimpanzee stone tools [15]; the latter techniques provide quantitative data of use-wear marks and descriptions that can be compared with archaeological pounding tools. Other research approaches have explored differences between the skills and mechanics used in nut cracking and stone tool knapping to assess the origins of flaking technologies [38, 39].

However, some comparisons of chimpanzee and archaeological assemblages are limited [40], as they compare intentionally knapped cores and flakes with pounding tools that have accidentally fractured. Such studies rarely have considered raw material differences between assemblages and their potential effect on the type and distribution of use-wear traces and development of fractures. It is more useful to compare chimpanzee stone tools with archaeological tools of the same raw material that have a similar function.

Pounding tools have been found in a number of Early Stone Age (ESA) archaeological sites [3, 41–43]. Identification of archaeological pounding tools relies on recognition of conspicuous marks, such as large battered areas, impacts, or specific fracture types [13, 43–46]. In places such as Olduvai Gorge, percussive processes were of greater importance than stone knapping in some assemblages, as indicated by the presence of a range of artefacts such as anvils, hammerstones, spheroids and subspheroids with battering marks and fractures linked to their use as percussive tools [13]. However, identifying the type of activity for which these objects were used is more problematic. At present, Gesher Benot Ya’aqov (Israel) is the only ESA site where it has been possible to link pounding tools to nut cracking, as the excellent preservation of the site has allowed identification of different nut species associated with pitted stones [47–49].

Although microscopic analysis has been widely used on flaked tools (following Semenov’s [50] work) and on grinding stones [51–55], it has rarely been applied to ESA stone tools [56–60]. To gain a better understanding of the role of percussive activities in human evolution, we have developed a systematic experimental approach. This approach uses a variety of analytical techniques, such as spatial distribution analysis and micro-wear studies [14, 61], and was used to analyse the pounding tools in this study.

## Materials and Methods

### Experimental settings

**Fig 1:**
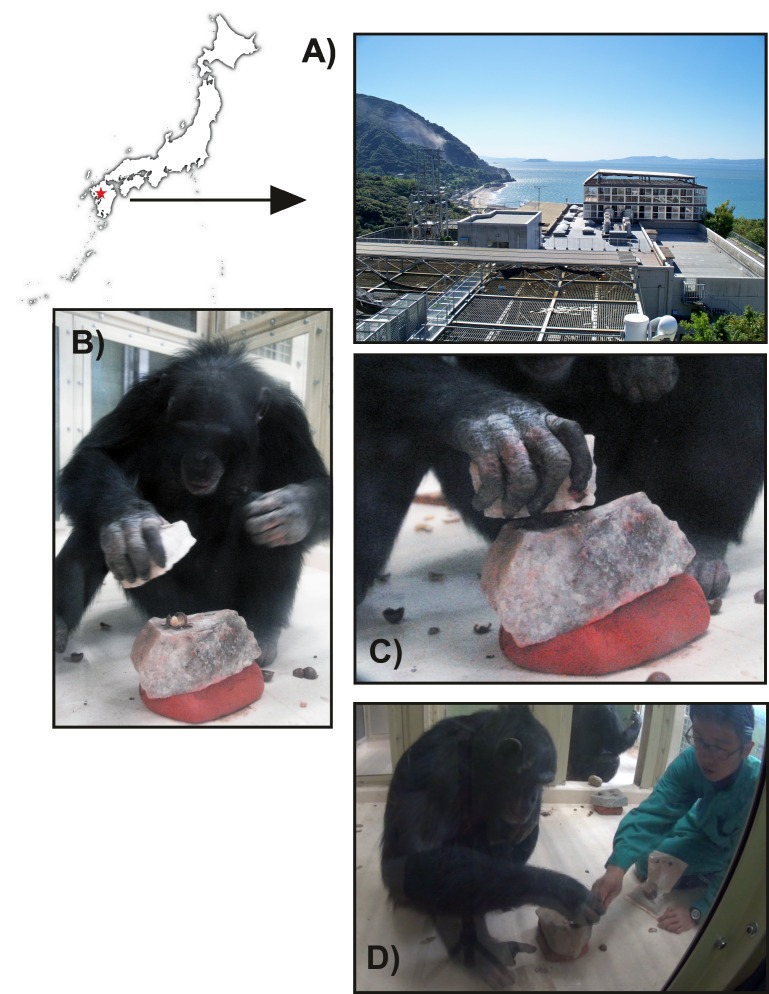
**Location and view of the Kumamoto Sanctuary (A); close-ups of the nut cracking experiments (B and C). View of the instructor (SH) and chimpanzee interacting during the experiments (D).** See S1 Video for a short video clip of the experiments.

Our experimental research took place at the Kumamoto Sanctuary Wildlife Research Center between July 26^th^ 2013 and October 17^th^ 2014 (Fig 1 and S1 Video). The individuals appearing on the videos and figures in this manuscript have given written informed consent (as outlined in PLOS consent form) to be shown. All experiments were conducted following protocols and ethical guidelines approved by the Animal Experimentation Committee of the Wildlife Research Center (Kyoto University). Four chimpanzees (*Pan troglodytes*) were selected based on their age and their previous experience with stone tool use: Tsubaki (female, 18 years; GAIN [Great Ape Information Network] #0551), Natsuki (female, 8 years; GAIN #0677), Mizuki (female, 16 years; GAIN #0559), and Kohtaroh (male, 21 years; GAIN #0500). Tsubaki, Natsuki, and Mizuki acquired the skill of using stone tools to crack nuts through social observation of conspecifics who had already mastered the technique, a process that has been described elsewhere [36, 37]. Once learnt, nut cracking became a regular activity among the chimpanzees, who were provided with nuts and could use stones available in their enclosure to crack them. Subsequently, these chimpanzees were included in other studies investigating the cognition underlying nut cracking behaviour [31, 62]. During the two weekly sessions, the three females (who had 5–10 years’ experience of the procedure) processed a total of 10–50 nuts.

The male chimpanzee Kohtaroh acquired the skill through active training by a human caretaker that began in November 2011. First, the caretaker trained the chimpanzee to use a stone tool by moulding: the caretaker gave the chimpanzee a hammerstone to grasp and then held the chimpanzee’s hand and moved it so that the hammerstone struck a nut that had been placed on a stone anvil. This type of training was provided about 10 times (i.e. 10 nuts cracked) in each session, two to five times per month, after which human intervention was gradually reduced until Kohtaroh could complete the entire process by himself. After a total of 32 training sessions over 19 months, Kohtaroh first accomplished the entire process alone in May 2013. The behaviour was encouraged through practiced one to three times per month until the present experiment began in July 2014.

Therefore, Mizuki, Tsubaki and Natsuki had some dexterity in nut cracking prior to the present study, and Kohtaroh had the least skill. However, Mizuki had trouble manipulating the tools during the experiments, owing to the tabular morphology of the hammer (and the way she held it and performed the striking movement). This made it difficult for her to successfully crack the nuts, so she was replaced with Natsuki. Natsuki and Tsubaki also had troubles handling the lava hammerstones due to their size.

The chimpanzees’ target was to strike a nut to access the kernel. As seen on the S1 Video, both hands are used for this activity but the hands do not make contact: one hand is used to hold the hammer and crack the nut while the other is used to place the nut on the anvil. The females always used their right hand when using the stone hammer, whereas the male always used his left. Hand preference emerged spontaneously, as occurs with wild chimpanzees.

The chimpanzee living areas were large and sufficiently complex to allow the animals to rest, exercise, and socialize with other members of the group [63]. They had three main meals per day of fresh fruits, vegetables, and nuts and leaves, supplemented with occasional enrichment programmes. Water was available throughout the day. The chimpanzees voluntarily participated in this study and were never deprived of food or water.

Nut cracking experiments took place in an enclosed indoor cage during feeding time. All sessions were recorded using three digital cameras (two located inside, and one outside the enclosure). The instructor placed the anvil on a terracotta slab to stabilize it and avoid damage produced by contact with the floor. A nut and hammerstone were placed close to the chimpanzee, whose task was to pick up the nut, place it on the anvil, and crack it with the hammerstone. Occasionally, the instructor helped the chimpanzees, especially when the nut rolled off the anvil surface, by repositioning the nut on the anvil. Experiments with Kohtaroh differed slightly, as the experiments were conducted through a mesh barrier for security reasons (S1 Video). In these sessions, the instructor placed the nut on the anvil and assisted Kohtaroh during the activity. The fact that Kohtaroh performed the experiments through a mesh barrier did not seem to be a constraint. Kohtaroh’s efficiency actually improved throughout the experimental sessions as he gradually required less strikes to open a nut and was able to crack nuts via correct axial movements without hitting the anvil.

To keep trials consistent, a maximum of 200 nuts was processed with each anvil and hammerstone. However, as shown in Table 1, it was not possible to complete sessions for one set of quartzite tools and two sets of lava tools due to handling problems (as described above).

**Table 1:** Summary of the stone samples used in the experimental sessions. The number of strikes per nut was obtained by dividing the total number of nuts cracked by the total number of strikes.

ID	Raw material	Individual	Total number of nut cracking sessions	Total nuts cracked	N Nuts per session	Total N Strikes	N Strikes per nut	Total time of use (in minutes)
O15-O35	Quartzite	Tsubaki	21	201	9.57	1053	5.24	107.14
O28-O50	Quartzite	Natsuki	12	201	16.75	1110	5.52	67.36
O40-O39	Quartzite	Mizuki / Natsuki	4	8	2.00	99	12.38	11.43
O48-O11	Quartzite	Kohtaroh	23	216	9.39	2536	11.74	136.33
O74-O128	Lava	Natsuki	3	9	3.00	73	8.11	8.56
O125-O72	Lava	Tsubaki	3	4	1.33	48	12.00	6.56
** **	** **	**Total**	**66**	**639**	**9.68**	**4919**	**7.70**	**337.38**

### Materials

We used macadamia nuts (*Macadamia integrifolia*) because the chimpanzees were familiar with them, and had processed them previously [36]. A macadamia nut has a diameter of approximately 23–25 mm, although it can reach 28 mm depending on the variety [64], and requires a compressive force of 2 kN to fracture [38]. Although we attempted during the experiments to introduce oil palm nuts (*Elaeis guineensis*), a species commonly consumed by African wild chimpanzees [24, 65–67], the subjects rejected them because of neophobia [68]. As macadamia nuts have a similar hardness to African nut species (*Elaeis guineensis* and *Coula edulis*), they are appropriate to model use-wear formation processes in the absence of African nuts.

Quartzite blocks (n = 8) and lava cobbles (n = 4) were used (see dimensions in Table 2) as hammers and anvils. Quartzite was collected from Naibor Soit (Olduvai Gorge), the primary source of quartzite used at Olduvai Gorge during the Pleistocene [69]. The lavas (basalt and trachyte) were mainly fine- and medium-grained cobbles collected from the river bed at Olduvai. All blanks were scrutinized before the experiments to document any pre-existing marks or fractures to avoid misidentification with use-wear marks, and were classified for use as active or passive elements (following Chavaillon’s terminology [70]).

**Table 2:** Basic measurements (in mm and g) of percussive artefacts. After the experiments, artefacts showed no visible modifications in size and only two lost mass owing to the detachment of fragments.

Tool ID	Function	N positives detached	Length	Width	Height	Weight	Weight difference
O15	Passive element	0	164	127	72	1904.5	-3.1
O35	Active element	1*	95	75	40	831.3	-1.7
O28	Passive element	0	190	140	85	3673.2	-0.6
O50	Active element	0	102	88	57	1121	0
O40	Passive element	0	150	95	55	1379.7	0
O39	Active element	0	115	70	42	963.2	0
O48	Passive element	0	232	92	74	3428	-4.6
O11	Active element	39	196	80	45	911.8	-45.6
O74	Passive element	0	114	96	30	451.3	0
O128	Active element	0	93	71	61	612.5	0
O125	Passive element	0	123	101	41	699.1	0
O72	Active element	0	99	62	39	358.8	0

*The positive detached from O35 was misplaced during the experiments.

This study followed protocols used by de la Torre et al. [14], and included a techno-typological description of pounding pieces as well as positives detached during their use. Low magnification use-wear analysis (<100×) was conducted using a Leica S8APO microscope with a magnification range of 1×–8×, equipped with a 10× lens, a 3.1-megapixel digital camera EC3, and fibre optic illumination. Spatial distribution analysis using GIS techniques was conducted. After the experiments, every rock sample was examined and the presence of residues and preliminary wear traces was recorded. The pieces were then rinsed with water and cleaned in an ultrasonic bath in neutral soap for 15–20 min to remove all residues, prior to detailed micro- and macroscopic analysis.

## Results and Discussion

### Techno-typological analysis

The same face of both anvils and hammerstones was used during the experiments to ensure consistency. Only in two instances (tool O35, used by Tsubaki) was the hammerstone accidentally turned over, but the use was negligible. In the case of O11 (used by Kohtaroh), the hammerstone plane was switched during the very first sessions at the beginning of the experiments, as Kohtaroh was having difficulties manipulating it. It was at this early stage of the experimental sessions that fragments were detached from the hammer.

All quartzite and lava samples showed little change to their original tabular and cobble morphology. Pieces O48 and O11 lost greater mass than other pieces (4.6 g and 45.6 g respectively), although their maximum dimensions remained the same. Objects O15 and O48 did not develop any fractures and the small amount of mass loss (Table 2) was related to the large crushed areas identified on their surfaces. Macroscopic traces on all quartzite samples were consistent, bearing small battered areas and impact points; only two pieces (O35 and O11) showed negatives caused by the detachment of percussive positives. Only two of the lava pieces showed superficial modification of their surfaces, represented by isolated impact points. The almost total absence of wear traces for lava hammers and anvils was related to their low degree of use (as shown in Table 1, it was not possible to complete the experiments with those tools, which were used to crack only 13 nuts in total).

**Fig 2:**
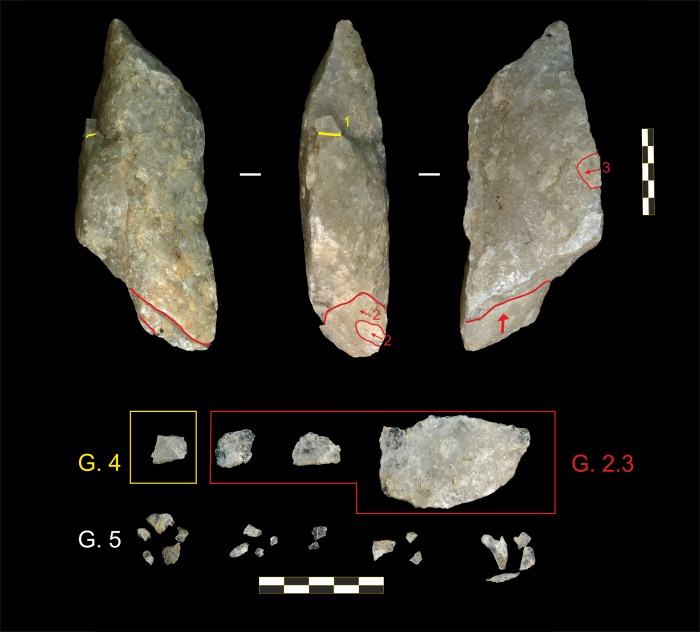
Percussive positives and refit from active element O11. See description of groups in Table 3 caption. All positives from Group 5 were detached during four separate nut cracking sessions. First, the positive from Group 4 (1), located on the left lateral side of the plane, was detached. This was followed by the two positives from Group 2.3 that conjoined on a dorsal-ventral refit located on the proximal plane (2). The last positive of the sequence is the fragment from Group 2.3 located on the right side of the horizontal plane (3), which was detached in a different experimental session.

Two pounding tools (O35 and O11, both used as active elements) developed fractures, although only fragments from O11 were recovered (n = 39) (Fig 2). Most positives were small fragments <15 mm long (Group 5; n = 35 [89.74%], see measurements in Table 3), one (2.56%) was an angular chunk (Group 4), and three (7.69%) showed characteristics typical of regular knapping flakes (Group 2.3); that is, possessing a butt, impact point, and bulb of percussion. Two fragments conjoined in a dorsal-ventral refit. The absence of battering traces on platforms indicates that these percussive positives were detached with a direct impact caused by contact between the active and the passive element.

**Table 3:** General measurements (in mm and g) of positives detached during the experiments. Group categories follow the classification established by de la Mora and de la Torre [13] and expanded by Arroyo [61]. Group 2.3: positives that mimic characteristics of knapping flakes having platforms, bulb of percussion, and impact point; Group 4: irregular fragments without any butt, bulb, or platform; Group 5: positives smaller than 20 mm on which features such as percussive marks, platforms, or impact points are absent.

		Minimum	Maximum	Mean	Std. Dev.
	Length	13	37	21.33	13.58
Group 2.3	Width	15	62	32.33	25.81
n = 3 (7.69%)	Thickness	3	11	6.00	4.36
	Weight	0.8	27.7	10.000	15.33
	Length	13	13	13.00	
Group 4	Width	11	11	11.00	
n = 1 (2.56%)	Thickness	6	6	6.00	
	Weight	1.4	1.4	1.400	
	Length	3	16	7.03	2.77
Group 5	Width	2	8	4.60	1.77
n = 35 (89.74%)	Thickness	1	5	1.54	0.95
	Weight	0.10	0.50	0.12	0.07

Most percussive by-products do not share the same morphological features as knapping flakes. A recent analysis of a sample of pounding tools used by wild chimpanzees from Bossou (Guinea) [61], identified edges, corners, and irregular fragments with the same morphological features as percussive positives recorded at the archaeological chimpanzee site of Noulo (Ivory Coast) [8] and, as those identified at Olduvai Gorge, which were originally classified as anvil fragments [13]. In all cases, percussive positives were produced mostly when the hammer came into contact with the anvil. Detachment is also greatly conditioned by raw material characteristics (isotropic rocks fracture more readily than anisotropic ones).

### Microscopic analysis

**Fig 3:**
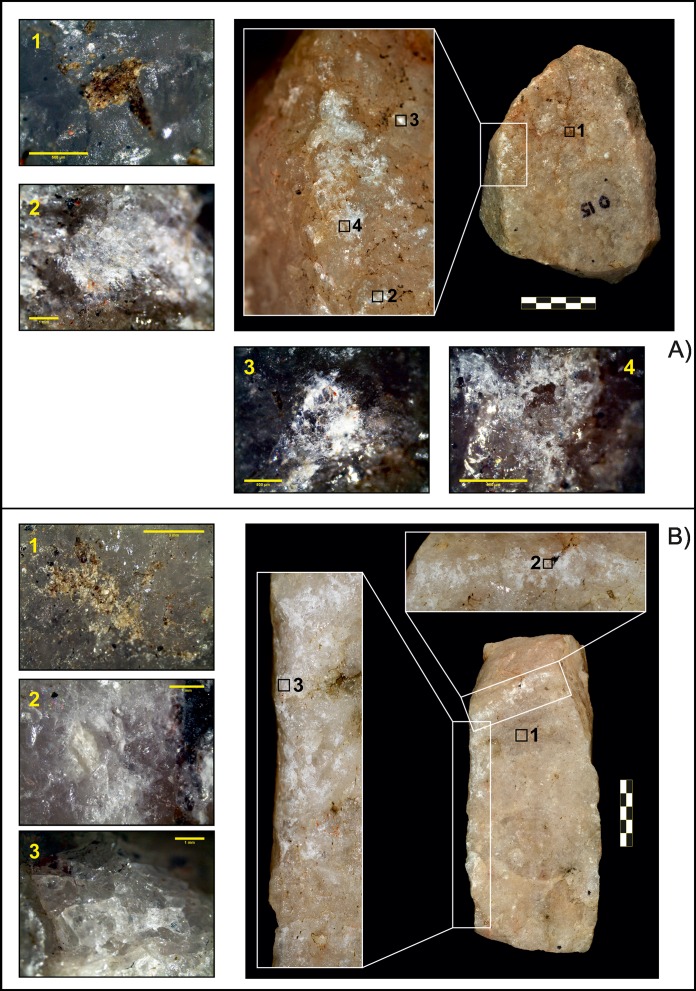
Quartzite passive elements. A) Piece O15 with crushing marks on the left lateral edge. 1. Residues identified before cleaning (80×, scale 500 μm); 2. Detail of crushing (20×, scale 1 mm); 3. Impact point (20×, scale 500 μm); 4. Pit associated with a crushed area (40×, scale 500 μm). B) Piece O48. 1. Detail of residues (16×, scale 3 mm); 2 and 3. Crushing (25× and 20×, both scales 1 mm).

Microscopic analysis of the pounding pieces revealed common patterns in both quartzite and lava objects. Isolated impact points with a circular shape were the main stigmas identified on the working surfaces of quartzite passive elements, as well as crushed areas resulting from a series of superposed impacts fracturing quartzite crystals (Table 4, Fig 3A and 3B). Occasionally, pits caused by micro-fracturing and detachment of small crystal fragments, were associated with these crushed areas (Fig 3A). Some residues were retained on the surface of areas where nuts were repeatedly placed. However, no modification of the blank could be identified in these areas after cleaning (Fig 3A and 3B).

**Table 4:** Summary of use-wear traces identified on the pounding tools used in the experimental programme.

	Percussive marks on peripheral areas							Percussive marks on central areas		
ID	Macro fractures	*Chipping*	*Crushing*	*Pits*	Impacts	Distribution	Incidence	Impacts	Distribution	Incidence
O15	x	X	√	x	√	Clustered	Medium	x	n/a	n/a
O35	√	√	√	x	√	Clustered	Medium	x	n/a	n/a
O28	x	X	√	√	x	Clustered	Low	x	Scattered	Low
O50	x	√	√	√	√	Clustered	Low	√	Scattered	Low
O40	x	X	x	x	√	Scattered	Low	√	Scattered	Low
O39	x	X	x	x	√	Scattered	Low	√	Scattered	Low
O48	√	√	√	√	√	Clustered	High	√	Scattered	Low
O11	√	√	√	x	√	Clustered	Medium	√	Scattered	Low
O74	x	X	x	x	x	n/a	n/a	√	Clustered	Low
O128	x	X	x	x	x	n/a	n/a	√	Clustered	Low
O125	x	X	x	x	x	n/a	n/a	x	n/a	n/a
O72	x	X	x	x	x	n/a	n/a	√	Scattered	Low

**Fig 4:**
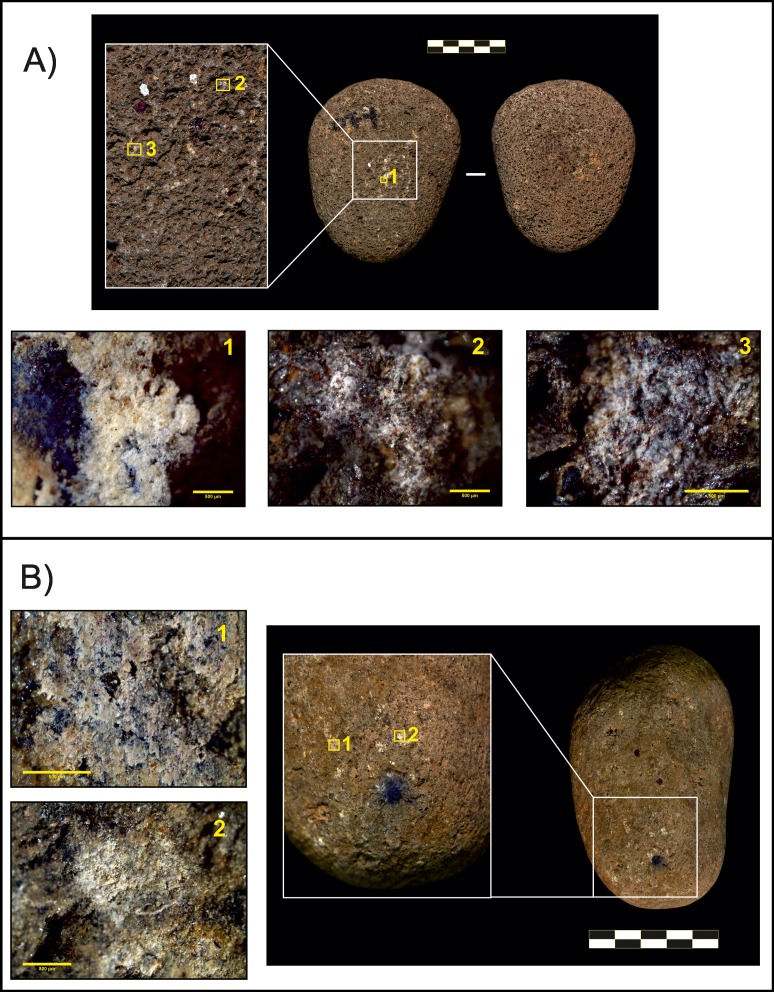
Lava pounding tools. A) Passive element O74. 1. Detail of residues (50×, scale 500 μm); 2 and 3. Impact points (50× and 80×, both scales 500 μm). B) Active element O72. 1. Residues on the working surface (80×, scale 500 μm); 2. Impact point (40×, scale 800 μm).

The main percussive traces identified on lava tools (both passive and active elements) were isolated, superficial impact points (Fig 4A), circular in morphology and with a diameter <1 mm. The absence of any visible mark on one tool (passive element O125) was related to a low degree of use. Residues were identified on the working surface of piece O74 (Fig 4A), enabling us to identify the area where nuts were placed. However, no microscopic traces had developed in this area.

**Fig 5:**
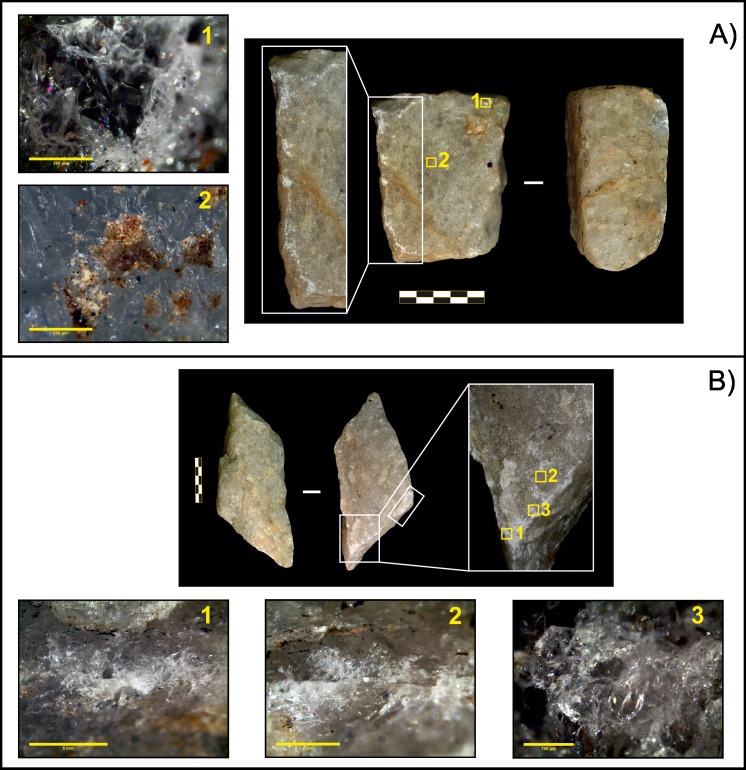
Quartzite active elements. A) Piece O35. 1. Detail of crushing associated with detachment of small crystal fragments (80×, scale 500 μm). 2. Residues (80×, scale 500 μm). B) Piece O11. 1 and 2. Crushing with associated microfractures (10× and 20×, scales 5 mm and 2 mm). 2. Impact point (50×, scale 700 μm).

Similar results were obtained in the analysis of active elements. All (four quartzite and two lavas) showed impacts on their surface, but only the three quartzite hammerstones used in completed sessions developed large crushed areas (a total of 4.87 cm^2^ for tool O11; 1.88 cm^2^ in O35; 1.24 cm^2^ for O50, S1 Table). Again, quartzite blanks showed a higher incidence of percussive traces (Fig 5) than lava blanks, as the latter were only used for a short period of time. Two pieces (O35 and O11) bore chipping marks on their edges associated with crushed areas, in which crystals appeared micro-fractured with occasional formation of hertzian cones associated with small detached fragments and the development of small pits.

### Spatial distribution of use-wear

Ten pieces developed clear macroscopic traces suitable for spatial distribution analysis following protocols set out by de la Torre et al. [14] (results shown in S1 Table). From a morphological perspective, damage affected a larger area on passive elements than active elements; the largest areas (>150 cm^2^) and perimeters (>50 cm) were on three anvils (O28, O48, and O15). The PA (percentage of tool surface covered by wear marks) and D (density of wear marks) indices allow us to quantify modification of the working surfaces.

Considering only those sets of tools with which the target of 200 nuts was reached (O15-O35, O28-O50, and O48-O11), GIS spatial analysis revealed that the highest percentages of surfaces covered by use-wear traces were found on the active elements with a mean PA of 2.51% (SD = 1.38%); the passive elements showed a mean PA of 1.94% (SD = 1.33%). A similar pattern was found for the density of wear traces: active elements showed the highest values (mean D = 0.13 cm^2^; SD = 0.05 cm^2^). Individually, the highest PA values were shown by the set of tools O11 and O48 (4.37% and 3.81%, respectively), which was used by Kohtaroh. Pieces O15 and O35 (used by Tsubaki) had the highest density of percussive traces (0.12 cm^2^ and 0.19 cm^2^, respectively), as this set showed the largest number of individual wear traces (S1 Table).

In contrast, two lava objects (active element O72 and passive element O74) had the lowest PA (<0.5%), but this low degree of modification was related to their low degree of use (Table 1). In general, all objects showed a very low D index regardless of their use; all values were always below 0.20% (S1 Table), indicating a low degree of modification on the quartzite and lava stones used by the chimpanzees.

**Fig 6:**
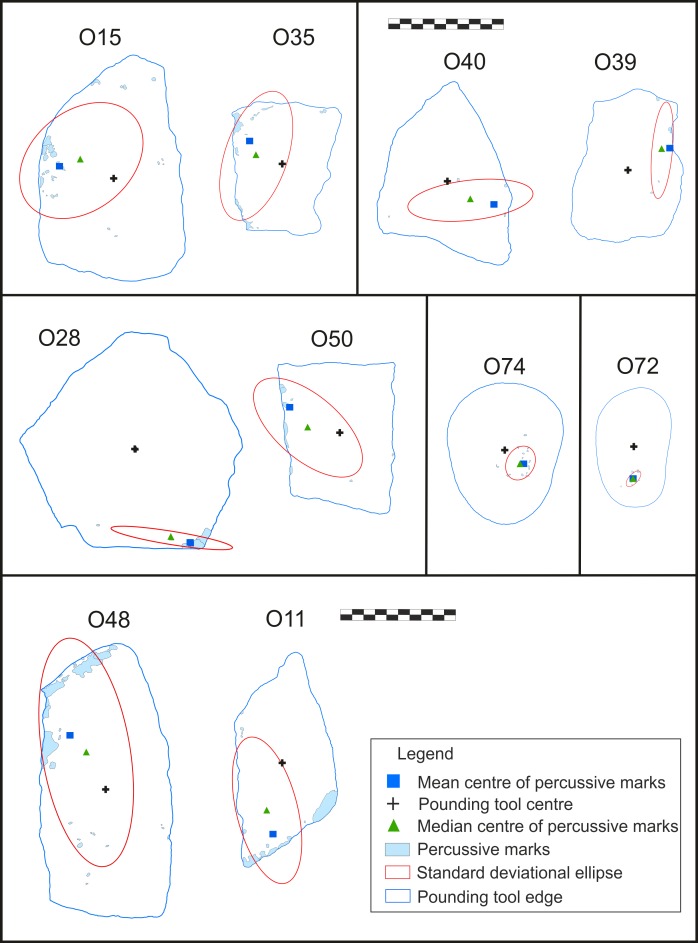
Spatial distribution analysis of pounding tools used in the experimental programme (see S1 Table for details). Analysis was not conducted on lava tools O74 and O128, as they did not develop macroscopic traces.

GIS analysis of distance to the anvil edge (DAE) and distance to the anvil centre (DAC) indicated that macroscopic wear traces were more frequently located close to the edges on all quartzite objects, regardless of their function or intensity of use. In contrast, these traces were more centred on lava tools (mean DAE <1.5 cm for all quartzite except piece O39, and between 2 and 3 cm for lavas; mean DAC >4 cm on quartzite samples and DAC <3 cm on lavas). The distribution of wear traces was more scattered on quartzite (mean elongation = 3.48; SD = 2.37) than on lava tools (mean elongation = 1.68; SD = 0.41) (Fig 6 and S1 Table).

In addition, analysis of the shape of percussive marks using the mean shape of percussive marks index (S1 Table) showed that, with the exception of one active element (O72) on which use-wear traces had a circular shape (mean shape of percussive marks = 1.04), traces on the percussive tools were more irregular in morphology.

### Use-wear formation processes

Wear traces on captive chimpanzee pounding tools may be associated with the contact between active and passive elements, but it is essential to understand why there is a general tendency for marks to be located close to the edge. To determine this, all video tapes were closely reviewed, allowing identification of three different mechanisms involved in the formation of use-wear traces (S2 Video).

**Fig 7:**
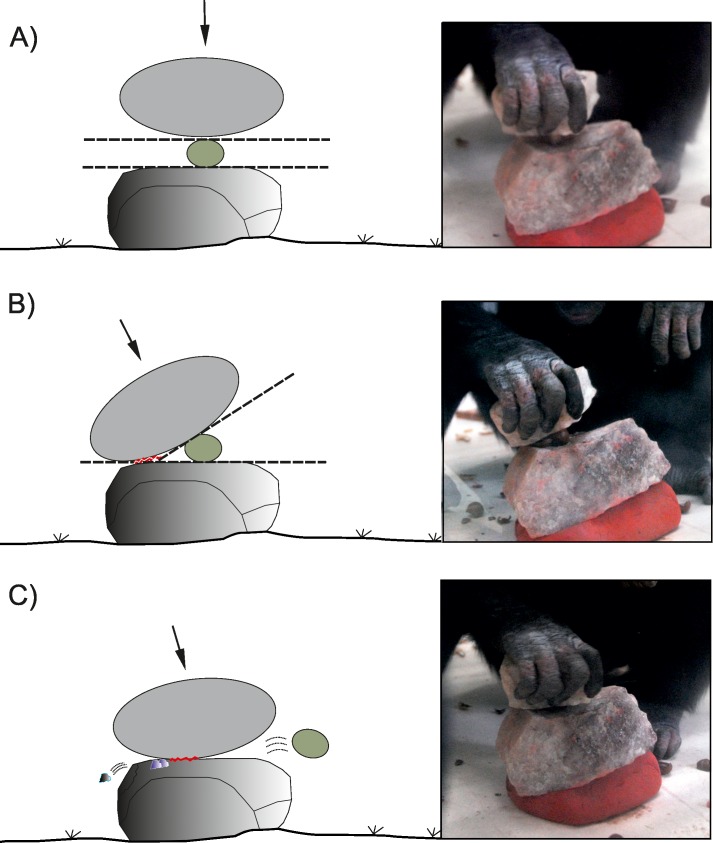
**Mechanisms that explain use-wear patterns identified on pounding tools used by captive chimpanzees**: A) Compression; B) Compression and peripheral contact; C) Direct impact.

The first mechanism is associated with the compressive force (Fig 7A) applied when an individual hits the nut using an axial movement. In this case, the active element is held perpendicular to the passive element and there is no contact between both pounding tools. As Olduvai quartzite and lava tools are hard and resistant raw materials, this compressive force does not produce any modification of the objects, because the force transmitted by the active element is absorbed mainly by the nut.

The second mechanism also involves an axial motion, and an occasional semi-circular movement. However, in this case the active element is handled at a 45° angle to the anvil, so that when hitting the nut, a compressive force is produced and peripheral contact between the active and the passive element occurs (Fig 7B). As a result, battered areas and concentrations of impact points appear in off-centre areas of the working surfaces.

The third mechanism is a direct impact, produced when the nut rolls away from the working surface or the subject fails to strike it, resulting in a direct hit on the passive element (Fig 7C).

The last two mechanisms may explain the development of fractures and percussive positives that were detached from the pounding tools, as well as the clear impacted and battered areas that developed across the active surfaces. Consequently, percussive traces described and identified during analysis of the experimental sample may be associated exclusively with a stone-against-stone process.

### Discussion

#### Comparison of captive and wild chimpanzee pounding tools

The results of the experimental programme described here can be compared with data collected from other analyses of wild chimpanzees. In this comparison, we focus on pounding tools, but it is worth noting that one of the main differences in nut cracking between wild chimpanzees and the captive chimpanzees in this study is how they execute the activity. In the wild, chimpanzees have to solve problems such as stabilizing the anvil or placing the nut on those areas of the anvil where it does not roll away. In the present experiment, the human instructor placed the passive element on a terracotta slab. This was to stabilize it and avoid damage produced by contact with the floor. In addition, when a nut rolled off the anvil, the human instructor assisted in repositioning it, to prevent the captive chimpanzee damaging the tools by hitting them against the floor and to guide the chimpanzee toward using the target anvil. In short, human assistance in the present study was aimed at simplifying variables so that we could focus on the effect of the impact on the hammer, anvil stone, and nut, and eliminate the effect of any contact between the anvil and the floor, or between the hammer and objects other than the anvil provided for the test.

All quartzite tools (both passive and active elements) used at the Kumamoto Sanctuary showed similar wear patterns, with impacts and small crushed areas located on peripheral areas of the active surfaces. Furthermore, there was a tendency for pounding tools used in nut cracking activities to have a low percentage of their surface covered by percussive marks (PA <5%).

There are some differences between the present spatial analysis results and the record of stone tools used by wild chimpanzees from Bossou. GIS analysis and 3D modelling of chimpanzee pounding tools at Bossou show that percussive marks are concentrated in central areas covering a large portion of the working surfaces, with the presence of off-centre percussive traces [15]. This pattern has been interpreted as indicating use by less skilled juvenile individuals and/or contact between the hammer and anvil [15].

We can now refine this hypothesis through analysis of the quartzite assemblage at the Kumamoto Sanctuary and the video-recordings data. The raw material, motion/kinetics, and individual skill level are the main factors that determine the formation and distribution of wear traces on chimpanzee stone tools. Friable and ductile raw materials tend to develop depressions, as observed on African iron oxide (laterite) anvils from Bossou [15] or wooden anvils, such as those found across the Tai forest in Ivory Coast [26], resulting in percussive marks that cover larger areas of the working surfaces. These depressions tend to grow in depth as anvils continue to be used, enhancing the stability of the nut. However, the compressive force applied when hitting a nut has considerably less effect on more resistant raw materials such as quartzite or basalt, so that macroscopically distinctive wear traces are produced only after recurrent use.

Kinetics plays an important role in successfully breaking the nutshell [31], and the motion and skill of individuals is important in the formation of percussive marks. In these cases, the way the subject manipulates the hammer and the manner in which the action is completed can influence the quantity and location of wear traces. This is especially so for tools with regular and hard surfaces (i.e. quartzite) on which the absence of depressions or irregularities makes placing the nuts more difficult, with the result that a larger area of the working surface is used.

Thus, at Kumamoto the pounding tools with larger areas of their working surfaces covered by percussive marks (PA >3.5%) were those used by the less skilled chimpanzee Kohtaroh, although the distribution pattern of use-wear does not differ from that of other percussive artefacts used by other individuals. At Bossou, however, identification of pounding tools used by juvenile or less skilled individuals is more difficult, as the same stones are reused by different individuals. Nevertheless, the clear presence of wear traces on peripheral areas and the greater number of fractures on tools, could be used as a reference point to detect the use of pounding tools by less skilled chimpanzees.

#### Comparison of chimpanzee and modern human experimental pounding tools

The distribution of percussive marks on the Kumamoto captive chimpanzee pounding tools are consistent with patterns recognized by de la Torre et al. [14] in a series of pounding experiments using Olduvai Gorge quartzite rocks for nut cracking, meat tenderizing, plant processing, bipolar knapping (in which a core is placed on an anvil and is struck with a hammerstone) and bone breaking. Their nut cracking experiments (processing *Coula edulis* and *Elaeis guineensis*) with modern humans also resulted in the production of off-centre isolated impacts on anvils caused by failed strikes.

On modern human and some captive chimpanzee nut cracking tools (O28, O50, O15), use-wear (PA index) covers less than 1.5% of their working surface (S1 Table, and de la Torre et al., Table 6 [14]), highlighting the low degree of modification caused by pounding tools and illustrating that force transmitted by the active element is absorbed mainly by the nut. Therefore, experiments undertaken by two different species with clear skill differences–for example, a modern human needed a total of 145 strikes to crack 1 kg of *Coula* nuts [14] (with an average of 2.2 hits per nut), whereas Tsubaki (the captive chimpanzee with the best score), required 5.24 hits to crack a macadamia nut–, showed that the same activity using the same raw material, produced similar wear trace patterns. These similarities can be explained from a mechanical perspective, because both humans and chimpanzees performed the activity using axial compressive force to break the nutshell [71].

**Fig 8:**
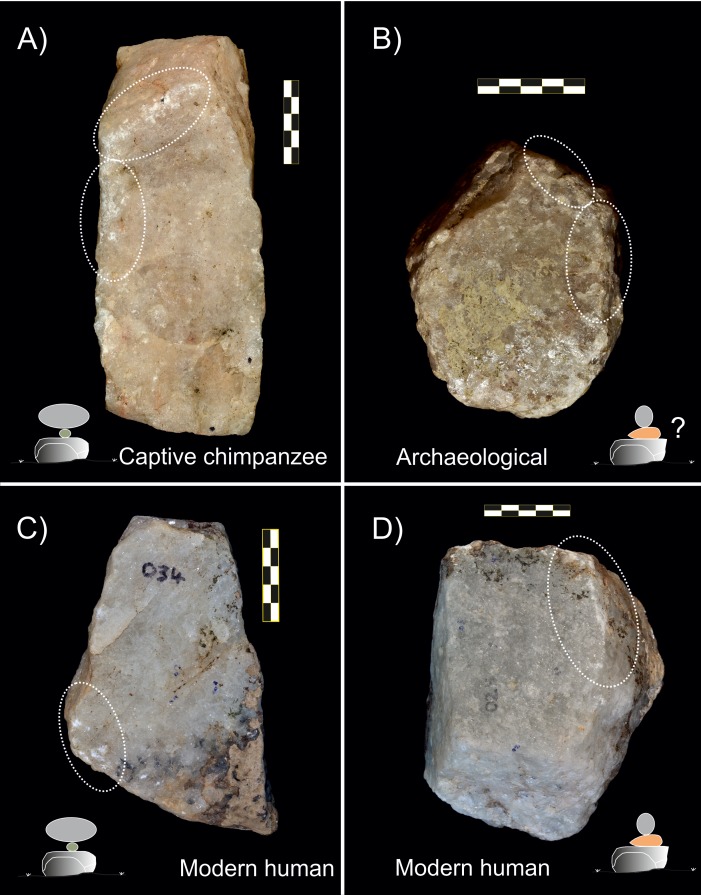
Comparison of different passive elements showing similarities in the wear pattern. A) Tool use by a captive chimpanzee; B) Anvil from SHK, Olduvai Gorge (Leakey’s collection); C) Anvil used to crack/open nuts by modern humans; D) Anvil used to tenderize meat (C and D from de la Torre et al., Figs 6B and 8C [14]).

It is also worth noting that wear patterns described on the sets of quartzite pounding tools used by captive chimpanzees also show similarities with some anvils used in meat and plant processing by modern humans [14] (Fig 8). Consequently, experimental quartzite pounding tools used by humans and chimpanzees have more similarities than differences, and the use-wear pattern on these tools encourages attempts to identify archaeological tools used for nut cracking. In addition, experiments with both modern humans [14] and captive chimpanzees (this study) indicate that some activities may leave very superficial impact marks, and suggest that many artefacts could have been used without showing apparent traces of modification.

#### Comparison with the archaeological record

One outcome of this study is the production of a reference collection of quartzite and lava pounding tools that can be compared directly with the archaeological record from Olduvai Gorge. Mary Leakey identified a large number of percussive artefacts in the Bed I and II assemblages [41], including anvils thought to be tools used in bone-breaking activities. Later analyses of the fossil assemblages showed the presence of percussive marks, suggesting that hominins were breaking bones to extract marrow [72, 73].

A techno-typological analysis of pounding tools from Olduvai Gorge by Mora and de la Torre revealed fractures, impacts, and battered areas visible macroscopically [13]. A recent microscopic analysis of a selection of pounding tools from Olduvai Gorge, originally classified by Leakey as anvils [74], showed percussive traces (mainly impact points and crushing areas), and occasional fractures located on the edges. A very low percentage of the surface (<1%, see [74]) of the so-called anvils classified by Leakey were covered by percussive traces, and had a similar wear pattern to that identified on anvils used by captive chimpanzees. Wear patterns on experimental anvils used in human pounding activities and archaeological pounding tools resemble those on tools used by the captive chimpanzees in this study.

More complex is the comparison between primate and archaeological active elements. The most common active elements in the archaeological record from Olduvai Gorge are hammerstones, which tend to be cobbles with traces of battering marks, and other types of pounding tools with different fractures; for example, hammerstones with fractured angles [13]. In our experiments, captive chimpanzees had problems manipulating lava cobbles because the cobbles were too small for the chimpanzees. Although only used for a short time, one cobble (O72, Fig 4B) showed a series of impact points that may replicate marks shown on some archaeological hammerstones (as these tools were used to crack less than 10 nuts and therefore had a very short use).

Nevertheless, chimpanzee active elements show a very low degree of modification (as seen on the spatial distribution section) compared with archaeological examples; they did not develop the large fractures found on archaeological active elements, suggesting that hominin hammerstones could have been used either for a longer period, or for another type of heavy-duty activity.

Direct comparisons of chimpanzee and hominin pounding tools tend to show a higher degree of modification of working surfaces on the latter. This characteristic may indicate that hominin tools were used in multiple tasks [13], whereas pounding tools used by captive chimpanzees were used solely to process nuts.

#### Do chimpanzees produce flakes?

It has been argued that, from a technological perspective, chimpanzees are able to obtain by-products similar to flakes found in the Oldowan record, which would support the correlation between chimpanzee nut cracking and the emergence of stone tool knapping [7, 11, 75, 76]. This theoretical model has been revised by Bril and colleagues [39], whose experiments show that stone tool knapping requires a higher level of cognitive capabilities than nut cracking (see [77] for a different point of view on the capabilities of hominins and chimpanzees). Other long-term experimental programs with bonobos (*Pan paniscus*) have focused on teaching individuals to produce and use sharp edges, but clear technological differences are evident when their flakes are compared with Oldowan assemblages [78, 79].

Despite metrical and morphological similarities between by-products detached from nut cracking tools and archaeological flakes, the main difference between both groups relates to the technological processes by which these flakes are produced (i.e. in flaking activities the knapper strikes the core to fracture it, whereas during nut cracking the target is the nut, not the anvil).

By-products detached from nut cracking tools are not the result of a deliberate process of conchoidal fracture and do not indicate an understanding of fracture mechanics or the intention to reduce a core to obtain flakes. Such characteristics can be recognized through a technological analysis of lithic assemblages in early Oldowan sites such as Gona [80] and Lokalalei 2C [43], and even in earlier stages of stone tool technology (the Lomekwian, [3]). In contrast, as some authors have emphasized [11, 12], by-products detached from chimpanzee nut cracking tools are caused by miss-hits or a process of repetitive contact between the hammer and anvil, and show no intentional flake removal.

The emergence of stone flaking is a major research question in human evolution. Researchers have suggested that pounding activities could have led to stone knapping [11–13]. However, it seems that between a pounding-like activity such as nut cracking and an intentional flaking activity there is an ‘intermediate step’, a stage in which the evolution of certain aspects related to the cognitive capabilities of hominins would have played an important role, and in which hominins would have acquired the skills necessary to systematically knap stone tools [2, 39]. However, this stage would be difficult to recognize in the fossil record because of the lack of archaeologically visible remains (but see Proffitt et al, [81]).

## Conclusions

This study examined pounding tools used by captive chimpanzees to process nuts, creating a referential framework that enable comparison with archaeological pounding tools. Our analysis has shown that passive and active elements used by captive chimpanzees tend to yield a similar wear pattern, especially on quartzite samples, which have crushed areas, impact points, occasional fractures located on areas close to the edges of the working surfaces, and a very low degree of modification. These wear traces are associated with three different mechanisms by which individuals manipulate the active element and, owing to the resistant properties of the raw materials, all stigmas can be associated with a stone-against-stone process, in which fractures that mimic features of knapped flakes are occasionally detached.

This is the first study to feature captive chimpanzee use of quartzite from Olduvai Gorge, and thus provides additional information about characteristics that can be used to identify pounding tools in the archaeological record and reconstruct hominin activities during the ESA. As one of the major problems in analysing an archaeological assemblage is the recognition of percussive activities, this reference collection provides an important comparative benchmark.

From an evolutionary perspective, the emergence of tool-using behaviour in hominins is an important research topic. Recent discoveries at Lomekwi 3 [3] and cut-marked fossils from Dikika (Ethiopia) [82] suggest that other non-*Homo* species were making and using stone tools. Studies of wild and captive chimpanzees offer a unique opportunity to model hominin behaviour. A better understanding of chimpanzee material culture could also aid interpretation of archaeological assemblages, highlighting the necessity for systematic comparisons between hominin and primate stone tools. Thus, future research should focus on increasing the sample of primate pounding tools, adding detailed descriptions (not only from wild chimpanzees, but also from other living non-human primates such as capuchin monkeys and long-tailed macaques), and expanding comparisons with different ESA pounding tools. Detailed comparative research programmes such as those described above, which combine aspects of archaeology and primatology, should help to improve our understanding of human evolution.

## Supporting Information

S1 Table Results of the geographic information system spatial distribution analysis conducted following protocols proposed by de la Torre et al. [14]. Abbreviations: PA: percentage of the area covered by use-wear; LUW: percentage of the area covered by the largest use-wear mark; D: density of percussive marks; ED: total perimeter of percussive marks divided by the total anvil area; MNSH: mean shape of percussive marks (comparing them with a standard circular shape, MNSH = 1). DAC: distance of percussive marks to the centre of the tool; DAE: distance of percussive mark to the edge of the tool; XstdD: standard deviation distance; EMNC-MDC: distance from the ellipse mean centre to the ellipse median centre; EMNC-AC: distance from the ellipse mean centre to the centre of the tool; EMNC-AE: distance from the ellipse mean centre to the tool edge.
(XLS)

S1 Video Overview of the experimental sessions with Tsubaki, Natsuki and Kohtaroh. (MP4)

S2 Video Detailed slow-motion view of mechanisms that explain use-wear formation on captive chimpanzee stone tools. (MP4)
